# Dynamic Response of Gradient Aluminum Foam Sandwich Tubes under External Explosive Loads

**DOI:** 10.3390/ma17184501

**Published:** 2024-09-13

**Authors:** Ting Li, Jiangping Zhao, Xuehui Yu, Anshuai Wang, Shangjun Chen, Na Ni, Zhushan Shao

**Affiliations:** 1 School of Resources Engineering, Xi’an University of Architecture and Technology, Xi’an 710055, China; liting@xauat.edu.cn; 2 School of Science, Xi’an University of Architecture and Technology, Xi’an 710055, China; 3 State Key Laboratory for Strength and Vibration of Mechanical Structures, School of Aerospace Engineering, Xi’an Jiaotong University, Xi’an 710049, China; 4 Department of Mechanics and Tianjin Key Laboratory of Nonlinear Dynamics and Control, Tianjin University, Tianjin 300350, China; 5 Xi’an Thermal Power Research Institute, Xi’an 710054, China

**Keywords:** 3D-voronoi technology, sandwich tube, density distribution, explosion resistance, numerical simulation

## Abstract

In this paper, we numerically investigate the dynamic response and explosion resistance of gradient aluminum foam sandwich tubes subjected to external blast loads. Based on 3D-Voronoi technology, we construct density-graded aluminum foam cores to systematically explore the influence of core density distribution, density gradient, and average relative density on the protective performance of these structures. Our primary objective is to identify optimal design parameters that maximize explosion mitigation capabilities while balancing energy absorption and specific energy absorption capacities. The research results show that a positive gradient core configuration exhibits superior anti-explosion performance, significantly outperforming its uniform and negatively graded counterparts, particularly when the gradient value is substantial. For the positive gradient cores, an increase in the gradient value leads to a corresponding enhancement in explosion resistance. Conversely, in negatively graded cores, a higher gradient value diminishes the anti-explosion performance. Furthermore, while augmenting the relative density of the core layer does improve the overall explosion resistance of the sandwich tube, it comes at the cost of reduced energy absorption and specific energy absorption capabilities, highlighting the need for a delicate balance among these competing factors.

## 1. Introduction

Aluminum foam, renowned for its remarkable properties, serves as a highly efficient energy-absorbing material. The material exhibits remarkable properties under compression conditions, including its lightweight properties, superior specific strength and specific stiffness, and long-lasting plateau stress performance [1,2,3,4]. An aluminum foam sandwich tube is a thin-walled structure composed of metal and an aluminum foam core; this material stands out for its remarkable capacity to absorb energy efficiently, ensuring high resilience against impacts while maintaining an impressively lightweight construction [5,6,7]. Numerous studies have shown that aluminum foam core tubes have better blast resistance compared with single solid tubes [8,9,10,11,12,13]. At the same time, when evaluating the anti-explosion performance of sandwich tubes, several crucial parameters [14] cannot be ignored, such as maximum displacement, total energy absorption capacity, and the specific energy absorption rate. These parameters together reflect the stability, energy dissipation efficiency and energy absorption efficiency per unit mass or unit volume of the sandwich tube under explosion shock. Consequently, the aluminum foam sandwich tube has gained extensive application in explosion protection scenarios.

At present, many researchers have conducted in-depth studies on the deformation mechanism and energy-absorbing properties of sandwich tubes under explosive loads using various methods such as experiments, numerical simulations, and theoretical analysis [15,16,17,18]. Karpenko et al. [19] proposed a method based on frequency response analysis, which successfully verified its accuracy and effectiveness in evaluating the dynamic properties of materials with nonlinear characteristics through detailed experimental research and advanced numerical simulation technology. Employing the Voronoi algorithm, Zhang and colleagues [20] developed a precise three-dimensional finite element simulation of a foam-cored structure. This model was specifically designed to capture the intricate deformation patterns exhibited by sandwich circular tubes filled with foam material under the influence of internal explosive loading. Their analysis demonstrated that the sandwich tube designed with a negative gradient core exhibited the best explosion-proof and impact resistance. In addition, they also deeply discussed the specific effects of explosive mass, inner and outer tube diameter and wall thickness, and core axial arrangement on structural dynamic response characteristics and energy absorption performance. Li et al. [21] studied the dynamic response of sandwich tubes under internal explosion loads by means of experiments and numerical simulations. They systematically studied the explosion process and structural response process of sandwich tubes, which was summarized as the following three stages: In the initial or first stage of the explosion, the explosion event breaks out from the starting point and continues until the generated shock wave or energy wave begins to interact directly and significantly with structures such as pipelines. In the second stage, the shock wave or energy wave generated by the explosion interacts directly and strongly with the pipeline and its accessories (fittings), resulting in significant deformation, displacement, or damage to the pipeline and its fittings. In the third stage, under the action of internal stress and kinetic energy generated by the explosion shock, because of the inertial characteristics of the material, the tube begins to undergo an autonomous deformation process directly driven by no external force.

To improve the energy absorption qualities of aluminum foam, research has been carried out based on uniform aluminum foam, resulting in gradient aluminum foam [22]. Sun et al. [11] deeply explored the performance of aluminum foam sandwich panels under explosion load. Their research conclusions clearly pointed out that when the core density of the sandwich panel showed a positive gradient distribution, its anti-explosion performance was significantly better than that of the sandwich panel with a negative gradient distribution of core density. Further examination revealed that as the disparity in the internal density of the core intensified, there were notable consequences, and the anti-explosion performance of the sandwich plate was also improved accordingly. Compared with sandwich plates with uniform-density cores, sandwich plates with large differences in core density and positive gradient distribution showed more excellent anti-explosion ability. On the contrary, sandwich panels with smaller density differences showed relatively weak anti-explosion performance. Utilizing both experimental and numerical simulation methods in conjunction, Jing et al. [10] explored the dynamic response characteristics of gradient aluminum foam sandwich plates under explosion load. They found that the positive gradient aluminum foam sandwich plate exhibited the highest specific energy absorption value compared with the other types, which meant that it could absorb and dissipate the energy generated by an explosion more effectively under the same conditions. The non-gradient sandwich plate followed, with the second-highest specific energy absorption performance. The negative gradient aluminum foam sandwich panel showed the lowest specific energy absorption value among the three, indicating that its energy absorption capacity was relatively weak under explosive impact. Based on the Split Hopkinson Pressure Bar (SHPB), Liu et al. [23] deeply studied the dynamic mechanical response and excellent energy absorption capacity of gradient aluminum foam materials under impact load. They found that gradient aluminum foam exhibited more excellent energy absorption performance than homogeneous aluminum foam. Specifically, the specific energy absorption rate of negative gradient aluminum foam was significantly higher than that of homogeneous aluminum foam. In addition, the failure modes of different gradient aluminum foams were observed and analyzed. The results showed that both the uniform aluminum foam and the positive gradient aluminum foam followed the failure mode of “from the proximal end to the distal end”, that is, the material at the initial end of the impact was destroyed first and gradually extended to the distal end. The negative gradient aluminum foam showed a failure mode of “from the far end to the near end”. Zhang et al. [24] used the numerical simulation method to deeply explore the dynamic response characteristics of the gradient sandwich plate under the impact of an underwater explosion. The simulation results showed that under the condition of maintaining the equivalent mass of the explosive as the control variable, the gradient sandwich plate exhibited better performance than the uniform sandwich plate—not only was the permanent displacement of the central region significantly reduced, but it also showed stronger energy absorption capacity. Although the deformation mechanism and energy absorption characteristics of gradient sandwich structures under explosive loading have been exhaustively explored in numerous studies, a scarcity of studies exists on the dynamic behavior of gradient aluminum foam sandwich tubes subjected to external explosive forces.

The aim of the present work is to study the numerical explosion resistance of sandwich tubes with density-graded aluminum foam under external explosive loads. In Section 2, the finite element modeling process of a sandwich pipe and the performance parameters of its constituent materials are discussed in depth. In Section 3, the accuracy of the finite element model of the sandwich tube is effectively verified by comparing the experimental results with the numerical simulation results. At the same time, the key parameters of the sandwich tube are described in detail. In order to further improve the accuracy and efficiency of the numerical simulation, grid sensitivity verification is carried out. In Section 4, numerical calculations are carried out, and the influences of the core density distribution, core density gradient, and average relative density on the dynamic response are discussed. After a thorough examination of the data and results, the definitive conclusions are outlined in Section 5.

## 2. Methods

### 2.1. Sandwich Tube Simulation Model

**Figure 1:**
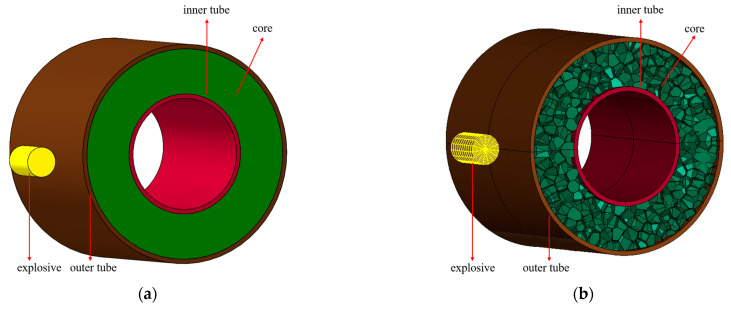
Diagrammatic representations of the aluminum foam sandwich tube structure: (**a**) 3D schematic and (**b**) finite element schematic.

The aluminum foam sandwich tube is composed of two thin-walled circular tubes filled with aluminum foam. The two thin-walled circular tubes are made of 45-gauge steel and are filled with two types of cores as follows: uniform aluminum foam cores and gradient aluminum foam cores. A uniform aluminum foam core indicates that the density of the aluminum foam is a fixed value, whereas a gradient aluminum foam core implies that the density of the aluminum foam varies with the change in the coordinate of the foam core layer. A schematic diagram of the aluminum foam sandwich tube is shown in Figure 1. Figure 1a shows a finite element diagram of the aluminum foam sandwich tube, while Figure 1b shows the 3D diagram of a sandwich tube.

#### 2.1.1. Sandwich Tube 3D-Voronoi Foam Model

The 2D-Voronoi model is widely used for its ability to simulate and analyze the mechanical properties of 2D honeycomb structures accurately. However, since aluminum foam is a three-dimensional material, its internal structure and property performance are more complex, so the 3D-Voronoi model must be adopted to describe the complex mesostructured of aluminum foam. The Voronoi configuration can be viewed as a variant of Tyson’s polygonal construction. A 2D-Voronoi structure emerges when a specific quantity of nucleation points is randomly dispersed in the plane and the perpendicular bisectors linking adjacent points are interconnected; in the three-dimensional space, a certain number of nucleation points are randomly generated, and the vertical bisectors of neighboring points are connected to form a 3D-Voronoi structure. To generate the 3D-Voronoi model, first, a total of N nucleation sites are randomly scattered throughout a volume V, and the distances between the neighboring nucleation points at different locations should be satisfied:

(1) $$\delta_{ij}\geq \delta_{ij}^{\min}$$

(2) $$\delta_{ij}^{\min}\;=\;(1-K)\delta_{ij}^{0}$$

where $\delta_{ij}^{\min}$ is the minimal distance existing between any pair of neighboring nucleation sites; K is the degree of irregularity defined by Zheng et al. [25], and K = 0.2 is taken here; and $\delta_{ij}^{0}$ is the distance between two neighboring nucleation points in the region, where its magnitude can be expressed as:

(3) $$\delta_{ij}^{0}=\frac{3(6+\sqrt{3})}{8}\frac{h_{0}}{\bar{\rho }[(x_{i}+x_{j})/2,(y_{i}+y_{j})/2,(z_{i}+z_{j})/2]}$$

where (x,y,z) are the coordinate values of the nucleation points of the cytosolic elements in the three directions, and $h_{0}$ is the cytosolic wall thickness of the generated model. Based on the above method, a series of gradient foam prototypes can be formulated, sharing a uniform average density and exhibiting a density gradient that follows a linear pattern. The model’s relative density profile can be formulated in the following manner:

(4) $$\rho (x)=\rho_{0}[1+k(z/H-1/2)]$$

where z is the direction in which the density gradient is located, $\rho_{0}$ is the average relative density of the model, k is the density of the model.

Based on the above method, the 3D-Voronoi foam model in the polar coordinate system is constructed, and the rectangular coordinate system is transformed into the following coordinates:

(5) x=r×cosθ

(6) y=r×cosθ

where r and θ are the radial and angular coordinates of the nucleation points in polar coordinates, respectively. N nucleation points are randomly distributed within a certain circular or doughnut volume, and the distance between neighboring nucleation points at different locations satisfies $\delta_{ij}\geq \delta_{ij}^{\min}$; $\delta_{ij}^{\min}=(1-K)\delta_{ij}^{0}$. The formula representing the relative density gradient of the 3D-Voronoi structure in polar coordinates is:

(7) $$\rho (r)=\rho_{0}[1+k(\frac{r-r_{1}}{R-r_{1}}-\frac{1}{2})]$$

where r is the direction in which the density gradient of the model lies, i.e., the radial direction in polar coordinates; R denotes the exterior diametric dimension of the ring; $r_{1}$ represents the interior diametric measurement of the ring; and $R-r_{1}$ is the length in the direction of the model density gradient.

**Figure 2:**
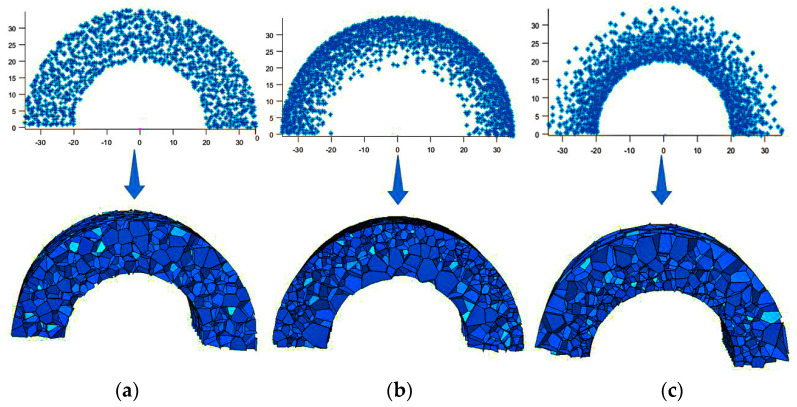
Scatter plot and 3D−Voronoi plot of an aluminum foam core layer with different density distribution modes. (**a**) A scatter diagram and 3D−Voronoi diagram of uniformly distributed cores. (**b**) A scatter diagram and 3D−Voronoi diagram of a core with positive gradient distribution. (**c**) A scatter diagram and 3D−Voronoi diagram of the a with negative gradient distribution.

Based on the relative density distribution of the 3D-Voronoi model in polar coordinates, a continuous density gradient foam core layer along the radius direction was established. The core layer density gradient types are the uniform (U) core layer, which means that the core layer density is equal everywhere; the positive gradient (P) core layer, which means that the density of the core layer is low near the inner diameter and high away from the inner diameter; and the negative gradient (N) core layer, which means that the density of the core layer is high near the inner diameter and low away from the inner diameter. Figure 2 depicts the distinctive structural features of three types of core layers including uniform, positive gradient, and negative gradient.

#### 2.1.2. Finite Element Model of Sandwich

**Figure 3:**
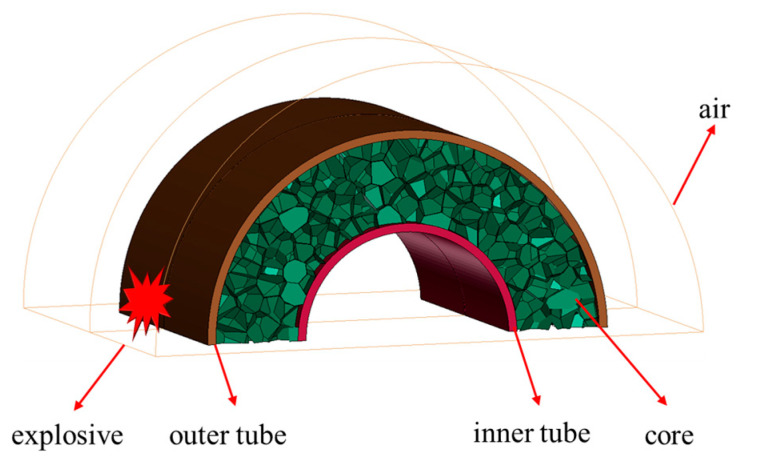
Finite element diagram of a 1/2 aluminum foam sandwich tube.

This study employed the ANSYS/LS-DYNA simulation tool to model the dynamic reactions of an aluminum foam sandwich tube during external blast loading. In order to simulate the accurate dynamic response of aluminum foam sandwich tubes under external explosion loads, different types of units were selected. Specifically, 8-node Solid164 solid elements were used for the air, inner and outer tubes, and explosives, while S3R and S4R shell elements were chosen to simulate the aluminum foam core. In order to reduce the amount of calculation, we took advantage of the symmetry of the aluminum foam sandwich tube and the explosion load, and chose to build a quarter model for simulation and analysis. Symmetric boundary conditions were applied to the two different cross-sections of the aluminum foam core tube. Symmetric boundary conditions were also imposed on two different cross-sections of air, and the rest of the surfaces were defined as non-reflection boundary conditions, in order to simulate a real explosion when explosives explode in the air and encounter non-reflection boundary conditions, ensuring the explosives wave continues to spread forward. In the numerical simulation, AUTOMATIC_SINGLR_SURFACE contact was employed between the foam aluminum cores, while AUTOMATIC_SURFACR_TO_SURFACE contact was adopted between the inner and outer tubes, as well as between the tubes and the cores. For all contacts involved, a friction coefficient value of 0.02 was assigned [26]. Utilizing the initial volume fraction method, the explosive material was introduced into the environment, and a fluid–structure interaction algorithm facilitated the simulations involving the aluminum foam sandwich tube, air, and explosive material [27]. The total computing time was set to 500 μs. A finite element diagram of a 1/2 aluminum foam sandwich tube model is shown in Figure 3.

### 2.2. Material Properties

The sandwich tube comprises inner and outer tubes, both fabricated from 45 steel material, which is sensitive to the strain rate. The Johnson–Cook (J-C) model was used as its constitutive model. The material parameters of the J-C model are shown in Table 1 [28].

Since aluminum foam is insensitive to the strain rate, MAT_PLASTIC_KINEMATIC was selected as the constitutive model in the simulation. The corresponding material parameters are shown in Table 2.

MAT_MULL was used as the constitutive model of air, and its density is 1.239 kg/m^3^. The state equation corresponding to the constitutive model is EOS_LINEAR_POLYNOMIAL, which is expressed as:

(8) $$p=C_{0}+C_{1}\mu +C_{2}\mu^{2}+C_{3}\mu^{3}+(C_{4}+C_{5}\mu +C_{6}\mu^{2})e$$

(9) $$\mu =\frac{1}{\upsilon }-1$$

where $C_{0}=C_{1}=C_{2}=C_{3}=C_{6}=0$ and $C_{4}=C_{5}=0.4$. The initial internal energy density $e_{0}$ is 2.5 × 10^5^ J/m^3^, and the initial relative volume $\upsilon_{0}$ is 1.

The explosion process of explosives is usually described by the EOS_JWL state equation in numerical simulation, and the corresponding pressure expression as:

(10) $$P=A(1-\frac{\omega }{R_{1}V})e^{-R_{1}V}+B(1-\frac{\omega }{R_{2}V})e^{-R_{2}V}+\frac{\omega E}{V}$$

In the pressure expression, the parameters A, B, $R_{1}$, $R_{2}$, and ω are constants, the parameter E signifies the initial specific energy content within the explosive material, and the parameter V designates the initial volumetric fraction of the explosive within a unit volume. The material parameters of the explosive are shown in Table 3.

## 3. Simulation and Parameters

### 3.1. Numerical Simulation Verification

#### Experimental Versus Simulation Outcomes: A Comparative Investigation

**Figure 4:**
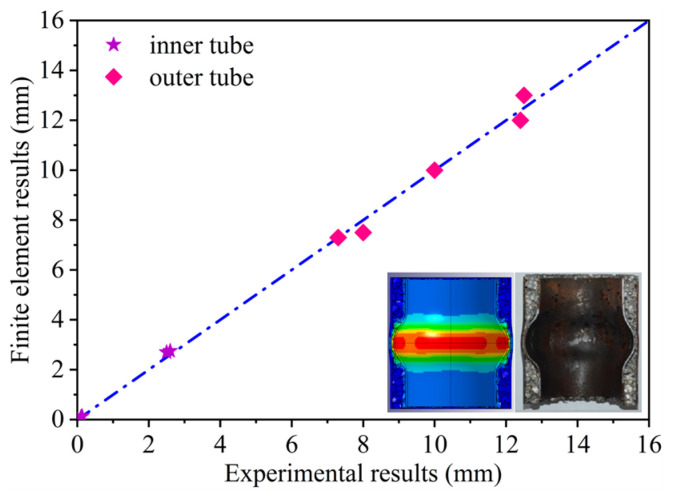
The comparison between finite element results and experimental results.

The finite element model developed in this study was evaluated for accuracy by comparing it to experimental outcomes reported in the literature [28]. As depicted in Figure 4, a thorough comparison reveals a substantial concordance between the outcomes of the finite element simulations and the experimental data, thereby robustly validating the precision and reliability of the finite element model constructed in this study.

### 3.2. Sandwich Tube Parameters

The specimen features an external tube diameter measuring 73 mm, an internal tube diameter of 37 mm, and a consistent wall thickness of 1.5 mm for both tubes. The thickness of the aluminum foam core in the sandwich tube is 15 mm, and its relative density is set to 11%. The length of the whole sandwich tube is 60 mm. The length–diameter ratio of the explosive is set to 1.5:1, and the initiation of the explosive is at a distance of 41.5 mm from the mid-span position of the specimen.

### 3.3. Mesh Sensitivity Verification

**Figure 5:**
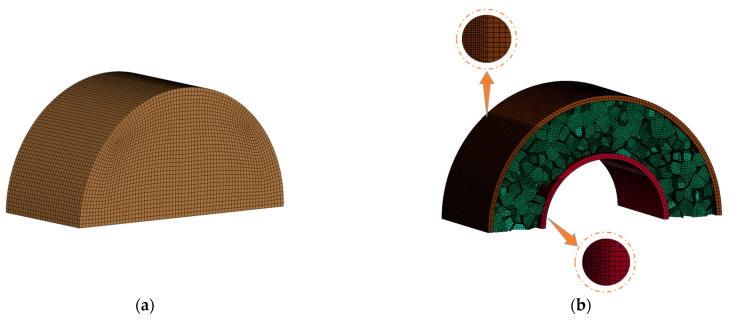
Grid division schematic diagram: (**a**) air and (**b**) sandwich tube.

**Figure 6:**
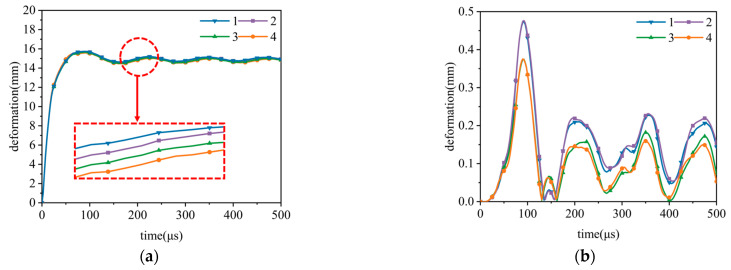
Deformation of the inner tube and the outer tube of the sandwich under different mesh sizes. (**a**) Deformation curve of the outer tube of the sandwich tube. (**b**) Deformation curve of the inner tube of the sandwich tube.

When performing the simulation studies described in this paper, the selection of the number of grids was crucial to ensure computational efficiency and computational accuracy. Therefore, in order to determine an economical and efficient mesh configuration, a mesh sensitivity validation analysis was performed for the finite element model. In this paper, four types of grids were analyzed, and the specific dimensions and number of each type of mesh are shown in Table 4. Figure 5 shows the detailed meshing of air and aluminum foam sandwich tubes. Figure 6 shows the deformation curves of the inner and outer tubes when the uniform aluminum foam sandwich tube is subjected to external explosion load under four different mesh sizes. The findings reveal minimal discrepancies in the deformation simulations of both the inner and outer tubes between the first and second grid configurations. After comprehensively weighing the requirements of computational efficiency and simulation accuracy, the parameters of the second grid type were finally selected as the grid parameters of the numerical simulation to guarantee the effectiveness and precision of this research.

## 4. Results and Discussion

To gain a more comprehensive understanding of the blast mitigation properties of the aluminum foam core sandwich tube under external explosive pressures, the influence of several key factors on its dynamic response was analyzed. These key factors include the density distribution, density gradient, and relative density of the aluminum foam core. This research endeavor explored 11 varieties of aluminum foam sandwich tubes, all of which were analyzed based on their varying specifications. The specific parameter values of these sandwich tubes are detailed in Table 5, where the first letter of the specimen number represents the meaning of the core, the second letter indicates the three different ways of distributing the density of the core, and the last letter and number represent the type of density gradient of the core as well as the specific value of the gradient.

Two important indicators in the evaluation of foam aluminum sandwich tube explosion resistance performance are the deformation of the inner and outer foam aluminum sandwich tube and the ability of each part of the sandwich tube to absorb energy. The specific energy absorption $E_{sa}$ is defined as the energy absorbed per unit mass of the structure and is given by [27]:

(11) $$E_{sa}=\frac{E_{a}}{M}$$

where $E_{sa}$ is the specific energy absorption, $E_{a}$ is the total energy absorbed by the sandwich tube, and M is the quality of the sandwich tube.

### 4.1. Dynamic Response of Aluminum Foam Sandwich Tubes under External Explosive Loading

**Figure 7:**
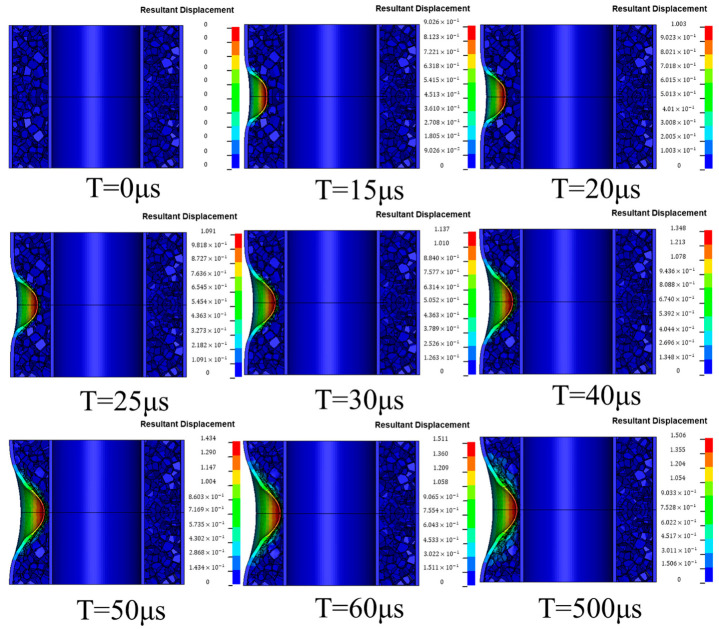
Deformation cloud diagram of the aluminum foam sandwich tubes.

Figure 7 shows the deformation cloud diagram of the uniform aluminum foam sandwich tube. The explosive wave first impacts the outer tube to deform it. Because of the deformation of the outer tube, the foam core also deforms and is gradually compacted under the combined action of the explosive wave and the core. After the core is compacted, pressure is applied to the inner tube, which leads to the deformation of the inner tube.

**Figure 8:**
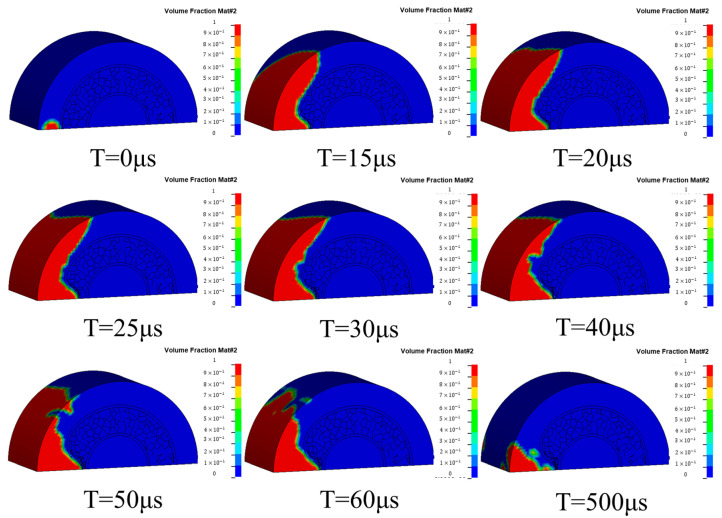
Propagation process of explosive waves.

Figure 8 shows the cloud diagram of the propagation process of the shock wave generated by the explosive explosion. It can be clearly observed in the diagram that when T = 0 μs, the explosive detonated instantaneously and began to release strong shock waves, which then began to spread outward. With the passage of time, when T = 15 μs, the fluid–solid coupling effect occurred between the shock wave generated by the explosive and the outer tube, which caused the outer tube to begin to deform and gradually crush the inner core. After T = 50 μs, it was obvious that the energy of the explosion shock wave gradually weakened, and the shock wave gradually attenuated.

### 4.2. The Influence of the Density Distribution on the Anti-Explosion Performance of the Sandwich Tubes

**Figure 9:**
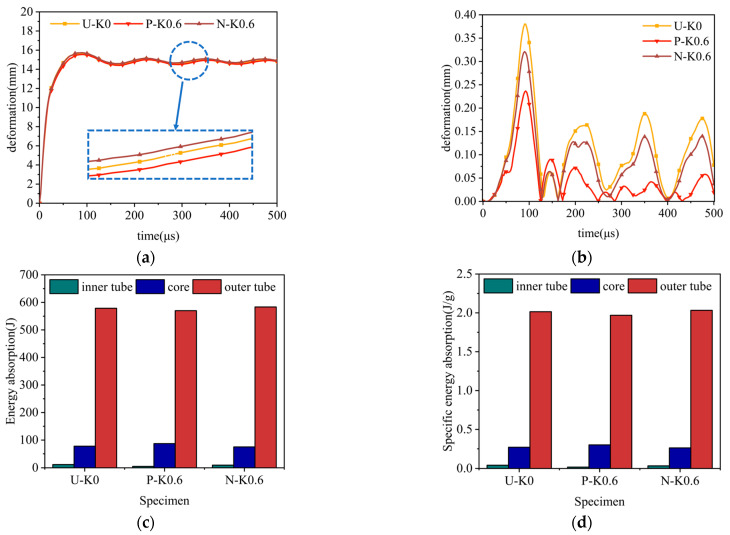
The effect of the core density distribution on the explosion resistance of the aluminum foam sandwich tubes when the core gradient is 0.6: (**a**) outer tube deformation; (**b**) inner tube deformation; (**c**) total energy absorption of the sandwich tube; and (**d**) specific energy absorption of the sandwich tubes.

**Figure 10:**
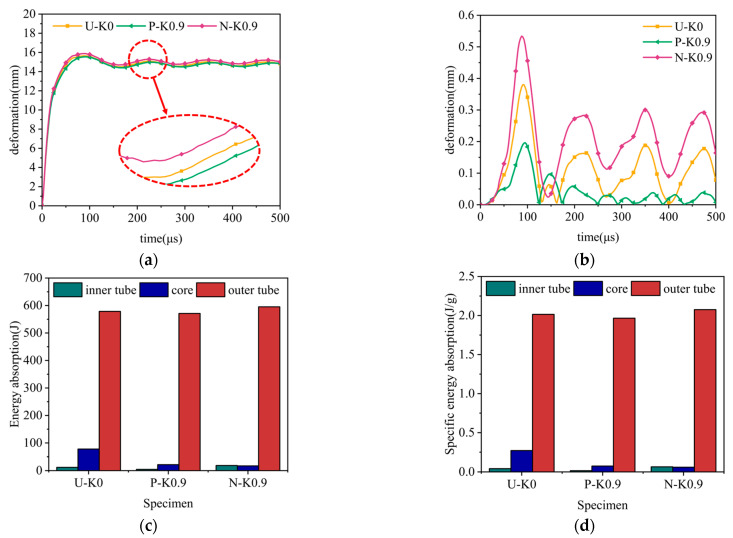
The effect of the core density distribution on the explosion resistance of the aluminum foam sandwich tubes when the core gradient is 0.9: (**a**) outer tube deformation; (**b**) inner tube deformation; (**c**) total energy absorption of the sandwich tube; and (**d**) specific energy absorption of the sandwich tube.

**Figure 11:**
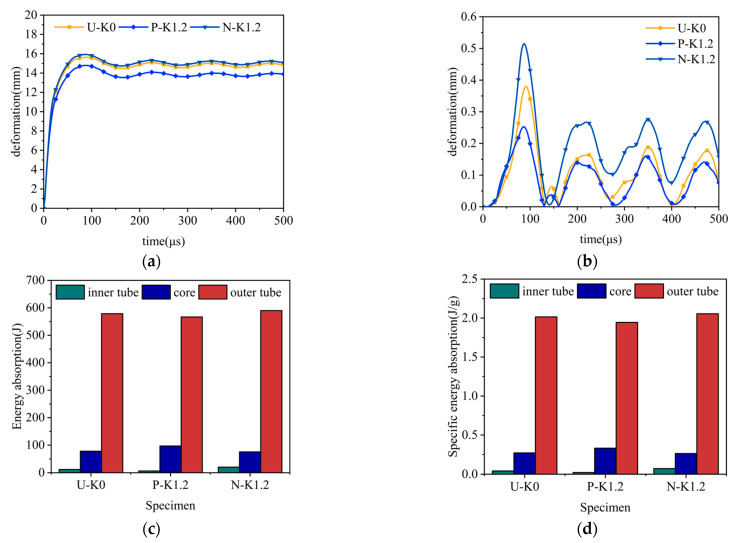
The effect of the core density distribution on the explosion resistance of aluminum foam sandwich tubes when the core gradient is 1.2: (**a**) outer tube deformation; (**b**) inner tube deformation; (**c**) total energy absorption of the sandwich tube; and (**d**) specific energy absorption of the sandwich tubes.

**Figure 12:**
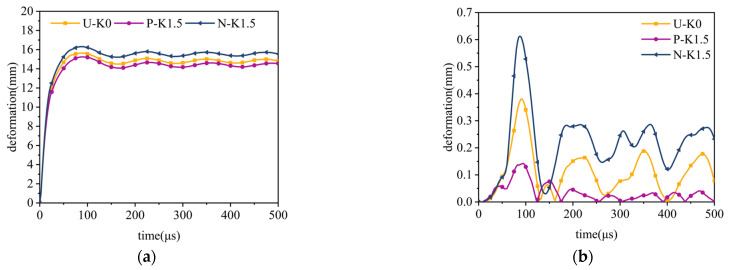

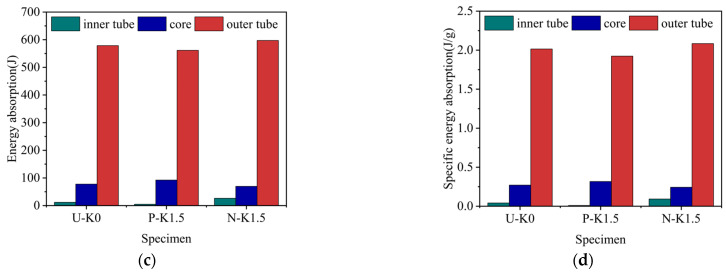
The effect of the core density distribution on the explosion resistance of the aluminum foam sandwich tubes when the core gradient is 1.5: (**a**) outer tube deformation; (**b**) inner tube deformation; (**c**) total energy absorption of the sandwich tube; and (**d**) specific energy absorption of the sandwich tubes.

Under constant explosion load, the deformation curves of sandwich tubes with continuous density gradient core are shown in Figure 9, Figure 10, Figure 11 and Figure 12. In addition, these diagrams describe in detail the energy absorption capacity and specific energy absorption rate of each component of the sandwich tube as a function of the core density gradient. When the core density gradient is 0.6 and 0.9, the outer tube’s deformation in the sandwich tube does not significantly vary. At core density gradient values of 1.2 and 1.5, based on the results, the outer tube’s deformation was ranked in terms of its severity, with N displaying the most significant deformation, followed closely by U and then P, with the least deformation. In particular, at a value of k equaling 1.2, the P-type sandwich tube exhibited minimal deformation in its outer tube, which was reduced by 5.44% in comparison with the U-type sandwich tube and by 7.11% in comparison with the N-type sandwich tube. Analyzing the deformation curves of the inner tube, we found that at k values of 0.6, 0.9, 1.2, and 1.5, the deformation of the inner tube was in the order of N > U > P from largest to smallest. It is particularly worth mentioning that when k = 1.5, the deformation of the inner tube of the P-type sandwich tube was the smallest, and the deformation was reduced by 62.57% in comparison with that of the U-type sandwich tube; compared with the N-type sandwich tube, it was reduced by 76.77%. Further analyzing the energy absorption and specific energy absorption data for five different density gradients for the core density distribution methods, we found that the changes in these values were not significant. Upon assessment of both energy absorption and specific energy absorption metrics, the P-type core surpassed both the U-type and N-type cores in terms of performance. Upon evaluating the deformation characteristics of both the inner and outer tubes, along with the energy absorption and specific energy absorption capabilities of the core, the P-type sandwich tube emerged as the superior choice for anti-explosion performance.

### 4.3. The Effect of Core Density Gradient Variations on the Resistance to Explosion of the Sandwich Tubes

**Figure 13:**
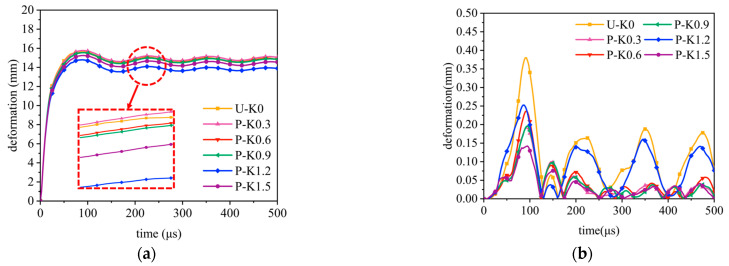

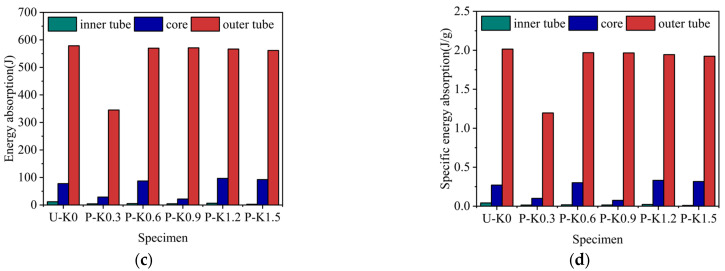
Anti-explosion performance of the aluminum foam sandwich tubes with a P-type sandwich tube: (**a**) outer tube deformation; (**b**) inner tube deformation; (**c**) total energy absorption of the sandwich tubes; and (**d**) specific energy absorption of the sandwich tubes.

Figure 13 shows the variation trend in deformation. The inner and outer tubes of the sandwich structure exhibit distinct capabilities in terms of both total and specific energy absorption under different density gradient values when the core density distribution is a P-type sandwich tube. In Figure 13a,b, it can be seen that when the k values are 0, 0.3, 0.6, and 0.9, the outer tube deformations of the aluminum foam sandwich tubes are relatively close to each other without significant differences. However, when the value of k is 1.2, the deformation of the outer tube of the aluminum foam sandwich tube decreases significantly and presents a minimum value. For the case of inner tube deformation, the deformation of the inner tube reaches the maximum when k = 0; on the contrary, the deformation of the inner tube is minimum when k = 1.5. At this time, there is a significant drop observed in the deformation of the inner tube compared with the U-type layer sandwich tube, with a decreased value of 62.57%. Based on the graphs of the absorbed and specific energy of the sandwich tubes, it is obvious that there is not much difference between the absorbed and specific energy of the aluminum foam core when the k values are 1.2 and 1.5. Compared with the case of the core energy absorption of the U-type layer sandwich tube, the absorption energy of the aluminum foam core at these two values of k increased by 24.38% and 18.77%, respectively. When evaluating the blast resistance performance of the sandwich tube, the small deformation of the inner tube, the core energy absorption, and the high value of the specific performance are the key measures. Considering the above data, we can conclude that the aluminum foam sandwich tube has the best blast resistance performance when k = 1.5.

**Figure 14:**
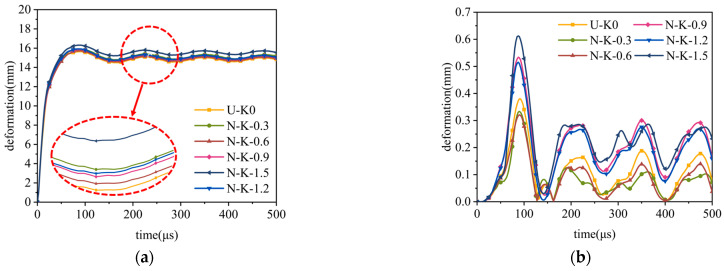

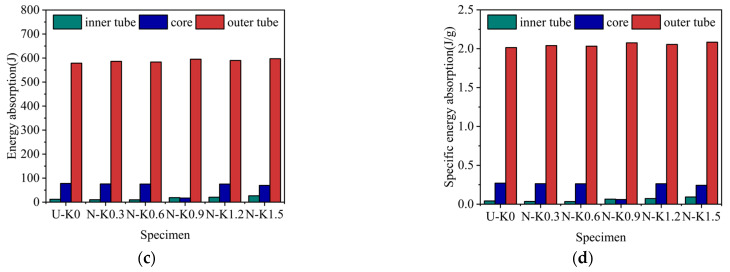
Anti-explosion performance of the aluminum foam sandwich tubes with an N-type sandwich tube: (**a**) outer tube deformation; (**b**) inner tube deformation; (**c**) total energy absorption of the sandwich tubes; and (**d**) specific energy absorption of the sandwich tubes.

Figure 14 depicts the alterations in distortion, energy dissipation, and energy absorption efficiency per unit mass of the inner and outer components of the N-type sandwich tube as a function of the density gradient parameter. The deformation of the outer tube of the sandwich tube reaches its maximum value when k = 1.5, while the difference in the deformation of the outer tube is not significant at the other values of the density gradient. It is particularly noteworthy that the outer tube’s deformation is minimized when the core’s density is distributed uniformly. Observing the deformation curve of the inner tube, we find that the deformation of the inner tube also reaches a maximum at k = 1.5, which indicates that the aluminum foam sandwich tube has the worst explosion resistance under this density gradient. When k = 0.3 and k = 0.6, the deformation of the inner tube of the sandwich tube does not differ much, but the deformation of the inner tube at k = 0.6 is relatively small, and the deformation is decreased by 15.5% compared with that of the U-type sandwich tube. By analyzing the energy absorption and specific energy absorption curves, we can see that when k = 0.6, the energy absorption as well as the specific energy absorption situation of the core shows an advantage and, at the same time, the energy absorption and specific energy absorption of the inner tube and the outer tube are also relatively good. Given the comprehensive consideration of the deformation of the inner tube of the sandwich tube, the absorption energy of the core, and the specific absorption energy situation, we can conclude that when k = 0.6, the sandwich tube exhibits superior performance in terms of its explosion resistance capabilities.

### 4.4. The Influence of Core Relative Density on the Anti-Explosion Performance of Aluminum Foam Sandwich Tubes

**Figure 15:**
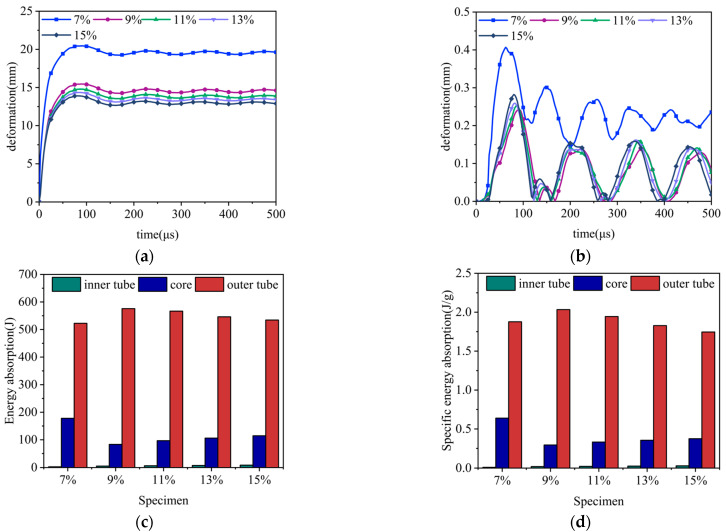
Anti-explosion performance of the aluminum foam sandwich tubes with a positive gradient core: (**a**) outer tube deformation; (**b**) inner tube deformation; (**c**) total energy absorption of the sandwich tubes; and (**d**) specific energy absorption of the sandwich tubes.

For the purpose of understanding the role of core relative density in enhancing the blast resistance of aluminum foam sandwich tubes, we conducted a detailed investigation, selecting five different density values under the condition that the core density was a positive gradient distribution and the gradient value was 1.2. Figure 15 shows the deformations of the inner and outer tubes as well as the changes in energy absorption and specific energy absorption of the aluminum foam sandwich tubes when subjected to external explosive loads at different core densities. Table 6 summarizes the key data points related to the specimens’ specific parameters, providing valuable insights into their characteristics. Figure 15a,b show the deformation of the inner tube and the outer tube with time under the external explosion load of the aluminum foam sandwich tube under different core relative densities. The aluminum foam sandwich tube’s energy absorption and specific energy absorption properties, as depicted in Figure 15c,d respectively, provide valuable insights into the behavior of its constituent parts under loading conditions. Examining the graphical representation of the data, it becomes evident that the deformation of both the internal and external tubes of the aluminum foam sandwich construction escalates markedly when the core’s relative density hits 7%. This marked increase in deformation signifies a relatively weaker blast resistance capability exhibited by the sandwich tube at this density point. In contrast, when the relative density of the core is 15%, although the deformation of the outer tube is the smallest, the deformation of the inner tube compared with the densities of 9%, 11%, and 13% is relatively large. It is noteworthy that at 11% and 13% core relative densities, the deformation variance between the inner and outer tubes is negligible, with both exhibiting comparable energy absorption capacities and specific energy absorption rates. Upon a thorough analysis of deformation in both the inner and outer sandwich tubes, alongside an assessment of energy absorption and specific energy absorption capabilities and economic considerations, the aluminum foam sandwich tube featuring a 11% relative density emerges as the optimal choice for the explosion protection project.

### 4.5. Overall Performance Comparison

**Figure 16:**
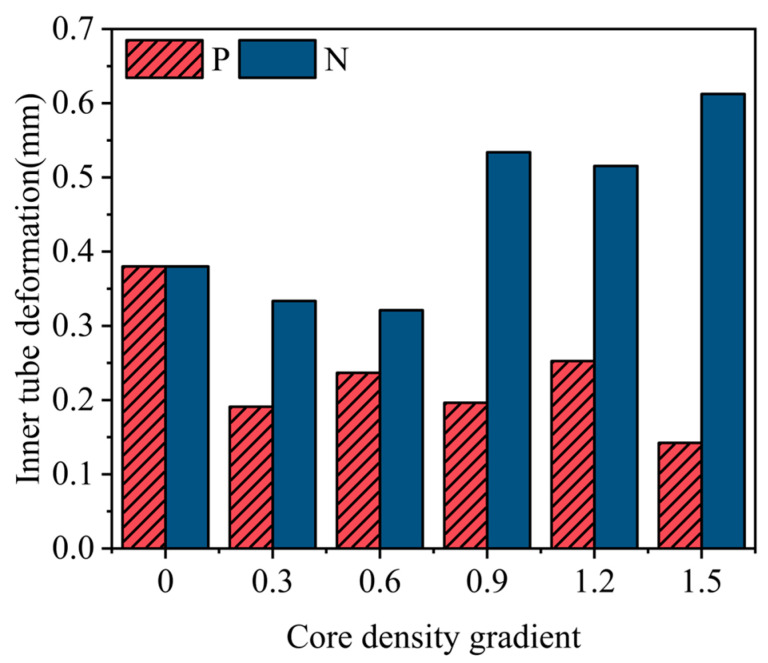
The overall comparison diagram of different density distribution modes.

Under the influence of external blast loads, the aluminum foam sandwich tube’s deformation behavior of its inner layer emerges as a pivotal factor in determining its effectiveness in mitigating explosion impacts and safeguarding lives. In order to compare the anti-explosive effect of three different density distributions including uniform aluminum foam, positive gradient aluminum foam, and negative gradient aluminum foam, we took the deformation of the inner tube as the basis for evaluation. As shown in Figure 16, the positive gradient aluminum foam sandwich tube exhibits a smaller inner tube deformation than the uniform aluminum foam sandwich tube at different gradients. This phenomenon indicates that the positive gradient aluminum foam sandwich tube is superior to the uniform aluminum foam sandwich tube in terms of blast resistance. The situation is different for the negative gradient aluminum foam sandwich tube. When the gradient value is less than 0.6, its inner tube deformation is less than the uniform aluminum foam sandwich tube, showing better resistance to explosion. However, once the gradient value exceeds 0.6, the deformation of the inner tube of the negative gradient aluminum foam sandwich tube exceeds that of the uniform aluminum foam sandwich tube, thus reducing its blast resistance. Based on the above analysis can conclude that in a specific range of gradient values, the negative gradient aluminum foam sandwich tube has better blast resistance than the uniform aluminum foam sandwich tube; but, once more than this range, its blast resistance will be inferior to the uniform aluminum foam sandwich tube. In contrast, the positive gradient aluminum foam sandwich tube shows better blast resistance than the uniform aluminum foam sandwich tube throughout the simulation.

## 5. Conclusions

A model for an aluminum foam sandwich tube was formulated utilizing the 3D-Voronoi technique, which was subsequently subjected to numerical simulations to evaluate its anti-explosion capabilities under external blast conditions. The effects of the density distribution, density gradient, and relative density of the aluminum foam sandwich core on its anti-explosion performance were analyzed. We drew the following conclusions:

(1) After a thorough comparison of the anti-explosion capabilities among uniform aluminum foam, positive gradient aluminum foam, and negative gradient aluminum foam sandwich tubes, it becomes evident that the positive gradient aluminum foam sandwich tubes consistently outperform their uniform counterparts, regardless of the specific gradient value employed. In contrast, for negative gradient aluminum foam sandwich tubes, their anti-explosion performance exhibits a nuanced trend: within a particular range of gradient values, they demonstrate enhanced resistance to explosions; however, surpassing this optimal range leads to a decline in performance, ultimately rendering them less effective than uniform aluminum foam sandwich tubes.
(2) The P-type sandwich tube exhibits a unique characteristic where the gradient value inversely correlates with the inner tube deformation; specifically, a higher gradient value leads to less deformation. However, this trend does not significantly impact the energy absorption or specific energy absorption of the core. Conversely, in the N-type sandwich tube, as the gradient value increases, the inner tube deformation also rises, accompanied by a downward trend in both energy absorption and specific energy absorption. A comparative analysis of the three sandwich tubes with varying density distributions clearly highlights the P-type sandwich tube as the superior performer in terms of anti-explosion capability.
(3) The crucial factor influencing the anti-explosion capability of the aluminum foam sandwich tube is the relative density of its core. Under identical explosion load conditions, an increase in the core’s relative density results in a corresponding reduction in the deformation of both the inner and outer tubes of the sandwich tube. However, this enhancement in structural integrity comes at the cost of decreased energy absorption and specific energy absorption capabilities of the core.

## Figures and Tables

**Table 1:** Material parameters of J-C model [28].

Material	Density (kg/m^3^)	Young’s Modulus (GPa)	A (MPa)	B (MPa)	n	c	m
steel	7850	210	507	320	0.28	0.064	1.06

**Table 2:** Material parameters of MAT_PLASTIC_KINEMATIC.

Density (kg/m^3^)	Young’s Modulus (GPa)	Poisson’s Ratio	Yield Strength (MPa)
2730	70	0.3	190

**Table 3:** Material parameters of the explosive [28].

Material	Density (kg/m^3^)	Detonation Velocity (m/s)	A (GPa)	B (GPa)	$R_{1}$	$R_{2}$	ω	E (GJ/m3)	V
JHL-3	1650	7050	611	10.7	4.4	1.2	0.35	8.9	1.0

**Table 4:** Parameters of different mesh sizes.

Program	Mesh Size (mm)	Mesh Number	Node Number
1	0.044	265,783	299,814
2	0.046	249,092	284,449
3	0.048	240,762	272,309
4	0.05	230,070	261,311

**Table 5:** Parameter values of the sandwich tube specimens.

Specimen Number	Inner Tube Diameter (mm)	Outer Tube Diameter (mm)	Inner (Outer) Tube Thickness (mm)	Core Thickness (mm)	Core Gradient	Core Density	Specimen Mass (g)	Specimen Length (mm)
C-U-K0	37	73	1.5	15	0	11%	287.21	60
P-K0.3	37	73	1.5	15	0.3	11%	288.58	60
P-K0.6	37	73	1.5	15	0.6	11%	289.46	60
P-K0.9	37	73	1.5	15	0.9	11%	290.46	60
P-K1.2	37	73	1.5	15	1.2	11%	291.59	60
P-K1.5	37	73	1.5	15	1.5	11%	291.96	60
N-K0.3	37	73	1.5	15	−0.3	11%	287.35	60
N-K0.6	37	73	1.5	15	−0.6	11%	287.07	60
N-K0.9	37	73	1.5	15	−0.9	11%	286.78	60
N-K1.2	37	73	1.5	15	−1.2	11%	287.10	60
N-K1.5	37	73	1.5	15	−1.5	11%	286.48	60

**Table 6:** Parameters of sandwich tube specimens with different core densities.

Specimen Number	Inner Tube Diameter (mm)	Outer Tube Diameter (mm)	Inner (Outer) Tube Thickness (mm)	Core Thickness (mm)	Core Gradient	Core Density	Specimen Mass (g)	Specimen Length (mm)
P-K1.2–7%	37	73	1.5	15	1.2	7%	278.34	60
P-K1.2–9%	37	73	1.5	15	1.2	9%	283.24	60
P-K1.2–11%	37	73	1.5	15	1.2	11%	291.59	60
P-K1.2–13%	37	73	1.5	15	1.2	13%	298.892	60
P-K1.2–15%	37	73	1.5	15	1.2	15%	306.198	60

## Data Availability

The data presented in this study are available on reasonable request from the corresponding author.
